# First-Principles Design of Qubits in Charged Carbon Nanomaterials

**DOI:** 10.3390/ma18112451

**Published:** 2025-05-23

**Authors:** Hongping Yang, Minghui Wu, Fengyan Xie, Dongli Meng, Jun Luo, Jing Zhu

**Affiliations:** 1 Fujian Key Laboratory of Functional Marine Sensing Materials, College of Materials and Chemical Engineering, Minjiang University, Fuzhou 350108, China; minghuiwu@mju.edu.cn (M.W.); xiefengyan517@163.com (F.X.); mengdongli@126.com (D.M.); 2 School of Materials Science and Engineering, Tianjin University of Technology, Tianjin 300384, China; jluo@tjut.edu.cn; 3 The State Key Laboratory of New Ceramics and Fine Processing and Key Laboratory of Advanced Materials (MOE), National Center for Electron Microscopy in Beijing, School of Materials Science and Engineering, Tsinghua University, Beijing 100084, China; jzhu@mail.tsinghua.edu.cn

**Keywords:** quantum computing, design of qubits, first-principles calculations, low-dimensional carbon nanomaterials

## Abstract

Our first-principles calculations have unveiled a profound influence of varied external charges on the energy levels and spin distributions of zero-, one-, and two-dimensional carbon nanomaterials. By leveraging the Fermi distribution formula, we systematically analyze the temperature-dependent electron occupancy probabilities of the highest occupied molecular orbital (HOMO) and lowest unoccupied molecular orbital (LUMO). Notably, configurations with specific additional electron loads exhibit a stable total occupancy of HOMO + LUMO equal to 1 across a wide temperature range, forming a robust basis for orbital qubits. This stability persists even under Fermi energy corrections, demonstrating minimal temperature sensitivity up to 300 K. Furthermore, we identify a universal criterion—E_HOMO_ + E_LUMO_ = 2E_Fermi_—that governs qubit feasibility across diverse carbon nanostructures, independent of dimensionality or atom count. Experimental validation via charge injection methods (e.g., gate modulation or electron beam irradiation) is supported by existing precedents in carbon-based quantum devices. Our findings establish low-dimensional carbon nanomaterials as versatile, scalable platforms for quantum computing, combining thermal stability and dimensional adaptability, thus bridging theoretical insights with practical quantum engineering.

## 1. Introduction

In recent years, as complementary metal–oxide–semiconductor (CMOS) integrated circuit technology has approached its technological limits, quantum computing has garnered increased attention as a promising next-generation computing paradigm [1,2,3,4,5,6,7]. Quantum computers represent a novel type of information processing system grounded in the principles of quantum mechanics. Due to their inherent parallelism, these systems have the potential to solve problems that are intractable for classical computers. For instance, the Rivest–Shamir–Adleman (RSA) algorithm, which forms the backbone of current public-key cryptographic systems, exemplifies an area where classical computers face exponential difficulty with increasing digit length [8]. On the contrary, quantum computers could efficiently factorize such large numbers using Shor’s algorithm in a very short time [9].

The quantum superposition is the cornerstone of quantum information theory, which enables a qubit to represent not only the classical states (0 and 1), but also any intermediate state. While a classical bit is restricted to either state 0 or 1, a qubit in the quantum realm can exist in a superposition of 0 and 1 simultaneously, or in any other combination of these states. Classical information processing is deterministic, relying on binary digits (bits), whereas quantum information processing is probabilistic, leveraging qubits that can be in any superposition state of 0 and 1. Quantum states are typically expressed using Dirac notation. A qubit is described by the superposition of two orthogonal basis states [10]:

|ψ〉 = α |0〉 + β |1〉(1)

Here, the probabilities of finding the qubit in states |0〉 and |1〉 are given by |α|^2^ and |β|^2^, respectively. The superposition principle dictates that α and β can be arbitrary complex numbers, as long as the sum of the squares of their magnitudes equals 1, i.e., |α|^2^ + |β|^2^ = 1.

A fundamental challenge in the development of quantum computers is identifying suitable physical systems that can serve as qubits. Over the years, a diverse array of qubit implementations have been explored, including spin qubits [11,12,13], photonic qubits [14], defect qubits [15], charge qubits [16], spin-photon qubits [17,18,19], nuclear-spin qubits [6,20], spin–orbit qubits [21], Wannier qubits [22], superconducting qubits [23,24], Josephson-junction qubits [25,26], and trapped ion qubits [1,3,27,28]. However, existing platforms face critical limitations. For example, superconducting qubits require cryogenic temperatures (<100 mK) to mitigate thermal noise, severely restricting scalability and operational flexibility; NV centers, while offering room-temperature operation, suffer from short coherence times due to spin–phonon coupling and challenges in deterministic placement; semiconductor quantum dots, though highly tunable, exhibit sensitivity to charge noise and fabrication variability. These limitations underscore the urgent need for alternative qubit platforms that combine thermal stability, scalability, and minimal decoherence.

Low-dimensional carbon nanomaterials—fullerenes, nanotubes, and graphene—emerge as compelling candidates. Their atomic-scale perfection, weak spin–orbit coupling, and dimensional adaptability offer unique advantages. For instance, graphene’s high carrier mobility and tunable bandgap [11], combined with the quantum confinement effects in nanotubes and fullerenes, enable precise control over electronic and spin states. Critically, these materials exhibit exceptional thermal stability, with spin lifetimes persisting up to room temperature, a feature unattainable in most conventional systems [29]. Despite these merits, a systematic framework for leveraging carbon nanomaterials as qubit platforms—particularly through charge engineering—remains unexplored.

In this work, we employed first-principles calculations based on density functional theory (DFT) to investigate the electrical and spintronic properties of graphene and carbon nanotubes under varying external charges. Our findings revealed that these materials exhibited distinct energy levels and spin distributions when subjected to different external charges. Specifically, for certain quantum dots, the highest occupied molecular orbital (HOMO) and the lowest unoccupied molecular orbital (LUMO) exhibited distinct spin configurations, such as being exclusively spin-up or a combination of spin-down and spin-up. Notably, in certain conditions, the combined electron occupation numbers of the HOMO and LUMO consistently summed to one, and the occupancy probabilities of these orbitals could be modulated by temperature. These findings herald a novel direction in quantum computing, as they underscore the potential of graphene and carbon nanotubes, endowed with tailored external charges, to serve as versatile orbital qubit platforms. This approach not only expands the toolkit for quantum information processing but also presents fresh avenues for exploring the fundamental physics underpinning quantum phenomena in nanoscale materials.

## 2. Models and Methods

First-principles calculations were performed using the Vienna ab initio Simulation Package (VASP) version 6.1.0 [30], employing the projector augmented wave (PAW) method [31]. The exchange-correlation energy was treated using the Perdew–Burke–Ernzerhof (PBE) functional [32], which is based on the generalized gradient approximation (GGA). A cutoff kinetic energy of 400 eV was established for the plane-wave basis set, and the convergence threshold energy was set at 10^−5^ eV. The atomic positions were fully relaxed until the force exerted on each atom fell below 0.01 eV/Å [33]. The supercell employed in the calculations featured a vacuum layer that exceed 15 Å (majority ≥ 20 Å) in all three dimensions, sufficiently large to preclude interactions between adjacent carbon nanomaterials, thereby necessitating only a single k-point to represent the Brillouin zone. The spin-polarized and non-spin-polarized states of all the models were calculated to determine their stability. The total energies were compared, and the lower-energy state was chosen as the ground state for the subsequent analyses.

**Figure 1:**
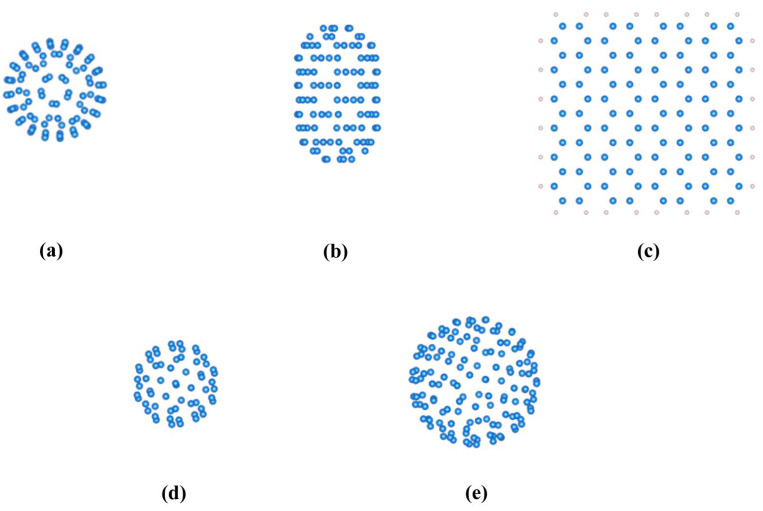
Models of carbon nanomaterials with varying numbers of carbon atoms (Carbon and hydrogen atoms are colored as blue and gray, respectively). (**a**) C_100_ fullerene (0D material, 100 C atoms). (**b**) (5,5) carbon nanotubes (1D material, 100 C atoms). (**c**) Graphene (2D material, 104 C atoms). (**d**) C_60_ fullerene (0D material, 60 C atoms). (**e**) C_140_ fullerene (0D material, 140 C atoms).

Figure 1 showcases a diverse array of carbon nanomaterial models, each featuring subtly varying C atom compositions. These include zero-dimensional nanomaterials, exemplified by C_100_ fullerene (Figure 1a), C_60_ (Figure 1d), and C_140_ fullerene (Figure 1e); one-dimensional systems, represented by (5,5) carbon nanotubes (Figure 1b); and a two-dimensional specimen, namely square graphene, where a consistent model with 104 C atoms (terminated by H atoms to mitigate dangling bonds) was utilized for comparison (Figure 1c). This comprehensive suite of models served as the foundation for our in-depth exploration into the electrical and spintronic properties of carbon nanomaterials.

In the realm of fullerene isomers, only one of those comprising 60 carbon atoms adhered to the isolated pentagon rule (IPR), denoted as model C_60_. Among the isomers with 100 carbon atoms, 450 met the IPR criteria, and we selected the lowest-energy isomer as the representative model (model C_100_) [34]. For fullerene isomers consisting of 140 carbon atoms, a total of 121,354 met the IPR standards. From these, we identified one model for each high-symmetry point group (I, D_5_, and C_3h_). After calculating the energies of these high-symmetry isomers, we chose the lowest-energy structure, C_140_-I, as the representative model (model C_140_). To validate the accuracy of the computational method employed in this study, we initially calculated the HOMO-LUMO energy gap of C_60_. Our theoretical result of 2.76 eV demonstrated excellent concordance with the 2.74 eV value documented in the literature [35], thereby attesting to the high accuracy of the calculation methodology utilized in this research.

## 3. Results and Discussions

### 3.1. One-Dimensional Carbon Nanomaterials Containing 100 Carbon Atoms with 0–6 Additional Electrons

**Figure 2:**
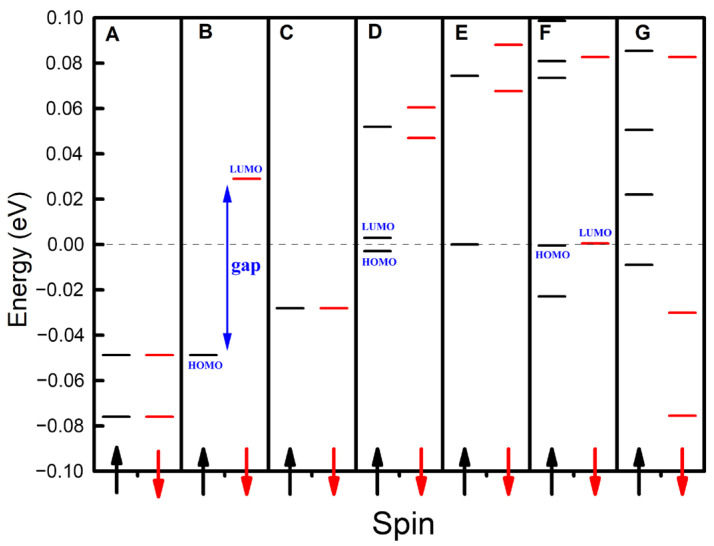
The distributions of the energy levels for the one-dimensional carbon nanomaterials using (5,5) carbon nanotubes. Panels A–G correspond to 0–6 additional electrons, respectively. The dashed line represents the Fermi level, while black energy levels and arrows denote spin-up configurations, and red energy levels and arrows indicate spin-down configurations.

As illustrated in Figure 2, we computed the energy level and spin distributions for one-dimensional carbon nanomaterials composed of (5,5) carbon nanotubes containing 100 carbon atoms, with panels A through G corresponding to 0 to 6 additional electrons, respectively. Due to spin polarization, the energy levels of these seven configurations split into spin-up and spin-down states, where the majority and minority spins were defined as spin-up and spin-down, respectively. Notably, each configuration exhibits a unique energy level and spin distribution profile, underscoring the sensitivity of these properties to even minute changes in electron count. Specifically, the highest occupied molecular orbital (HOMO) and the lowest unoccupied molecular orbital (LUMO) of Configurations B and F were spin-up and spin-down, respectively. In contrast, the HOMOs and LUMOs of Configurations D, E, and G were both spin-up. The spin-dependent bandgap arises from spin-polarized charge redistribution under external fields. Spin-up/down channels experience distinct electrostatic potentials due to Pauli exclusion, lifting degeneracy in frontier orbitals. This effect is amplified in low-dimensional systems with strong quantum confinement [12,34]. These findings underscore the potential of these one-dimensional carbon nanomaterials to serve as tunable platforms for manipulating spin distributions and generating electronic currents with tailored spin polarizations, a capability that holds immense promise for applications in spintronics.

**Figure 3:**
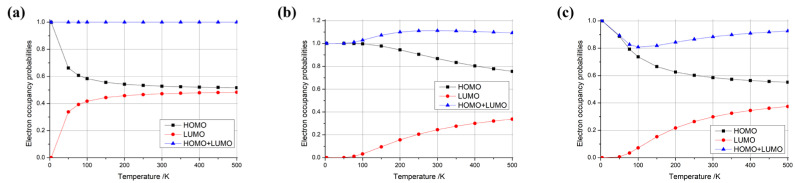
Typical temperature-dependent electron occupancy probabilities for HOMO, LUMO, and their combined (HOMO + LUMO) states in (5,5) carbon nanotube-based nanomaterials. Cases (**a**–**c**) showcase distinct behaviors observed in systems with three, one, and six additional electrons, respectively.

Initially, we assumed that the energy levels remain constant with changes in temperature. The electron occupancy probability at a given energy level can be described by the Fermi distribution formula [36]:

(2) $$f(E)=\frac{1}{e^{\frac{E-E_{F}}{k_{B}T}}+1}$$

where $k_{B}$ is Boltzmann’s constant, *T* is the temperature, $E_{F}$ is the Fermi energy, *E* is the energy of an individual level, and $f\left(E\right)$ is the occupancy probability of the level. Using this formula, we calculated the electron occupancy probabilities of the highest occupied molecular orbital (HOMO) and the lowest unoccupied molecular orbital (LUMO) at different temperatures. We observed three typical distributions for the difference in HOMO + LUMO occupancy probabilities with temperature, as shown in Figure 3, where Figure 3a depicts the case where the occupancy probability of HOMO + LUMO is always equal to 1, Figure 3b shows the case where the occupancy probability of HOMO + LUMO is always greater than 1, and Figure 3c illustrates the case where the occupancy probability of HOMO + LUMO is always less than 1.

**Figure 4:**
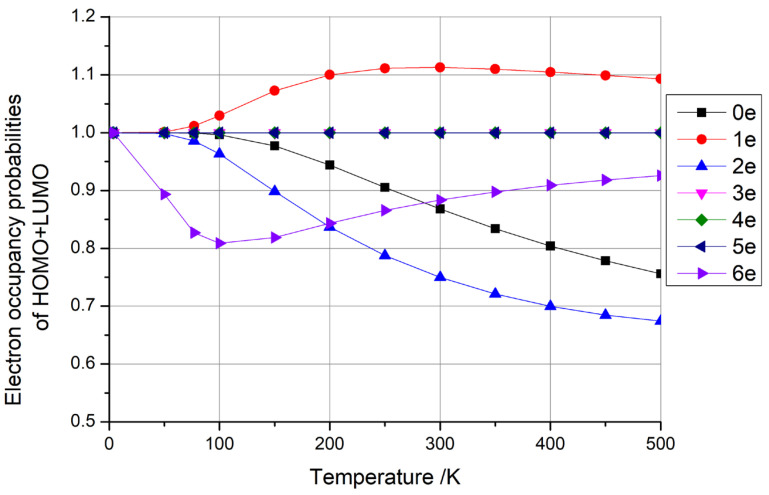
The electron occupancy probabilities of HOMO + LUMO as functions of temperature for one-dimensional carbon nanomaterials comprising (5,5) carbon nanotubes with 0–6 additional electrons, respectively.

In particular, when considering the type depicted in Figure 3a (HOMO + LUMO = 1), as illustrated in conjunction with Figure 4 (with three, four, or five additional electrons), the HOMO occupancy probability decreased monotonically from 1 to 0.5 as the temperature increased. Conversely, the LUMO occupancy probability increased monotonically from 0 to 0.5 with rising temperature. However, the sum of the HOMO + LUMO occupancy probability remained constant at 1. This indicates that the electron’s occupied state is a superposition of the HOMO and LUMO, with the sum of the probabilities of these occupied states remaining invariant at 1, which can be considered as a qubit. This electron possessed the highest energy within the system, enabling it to effortlessly migrate to other locations for subsequent operations. Furthermore, with the provision of an electron source, a steady stream of electrons in distinct, specific superposition states could be generated by adjusting the temperature. This constitutes a groundbreaking, innovative concept in the realm of quantum computer design.

Then, considering the type depicted in Figure 3b (HOMO + LUMO > 1), as illustrated in conjunction with Figure 4 (with one electron), the sum of the occupancy probabilities of HOMO + LUMO initially began at 1, increased with rising temperature, and then decreased with further temperature increases. During this phase, the sum of the occupancy probabilities was always greater than 1, rendering it unsuitable for use as a qubit. Similarly, considering the type depicted in Figure 3c (HOMO + LUMO < 1), as illustrated in conjunction with Figure 4 (with zero, two, or six electrons), the sum of the occupancy probabilities of HOMO + LUMO also started at 1, decreased with rising temperature, and then increased with further temperature increases. In particular, the anomalous behavior observed in carbon nanotubes with six additional electrons arises from the relative alignment of the HOMO/LUMO energy levels with the Fermi level. Similarly to the scenario depicted in Figure 3c, when the carbon nanotube is charged with a specific electron count, the occupancy of the HOMO decreases monotonically with rising temperature, while the LUMO occupancy increases. At lower temperatures, the HOMO occupancy decays more rapidly than the LUMO occupancy rises, leading to a minimum in the combined HOMO + LUMO occupancy (e.g., at ~100 K for the six-electron case). For other charge states, the summation of HOMO + LUMO occupancies exhibits distinct behaviors depending on the relative alignment of the HOMO/LUMO levels with the Fermi level, resulting in divergent Fermi–Dirac distribution profiles. Consequently, the sum of the occupancy probabilities was always less than 1, making these configurations unsuitable for use as qubits.

Previously, we assumed that the Fermi energy remained constant with temperature changes; however, the Fermi energy is expected to vary slightly with temperature according to the Fermi energy correction formula [37]:

(3) $$E_{F}=E_{F}^{0}\left[1-\frac{\pi^{2}}{12}{\left(\frac{k_{B}T}{E_{F}^{0}}\right)}^{2}\right]$$

where $k_{B}$ is Boltzmann’s constant, *T* is the temperature, $E_{F}^{0}$ is the original Fermi energy, and $E_{F}$ is the corrected Fermi energy.

**Figure 5:**
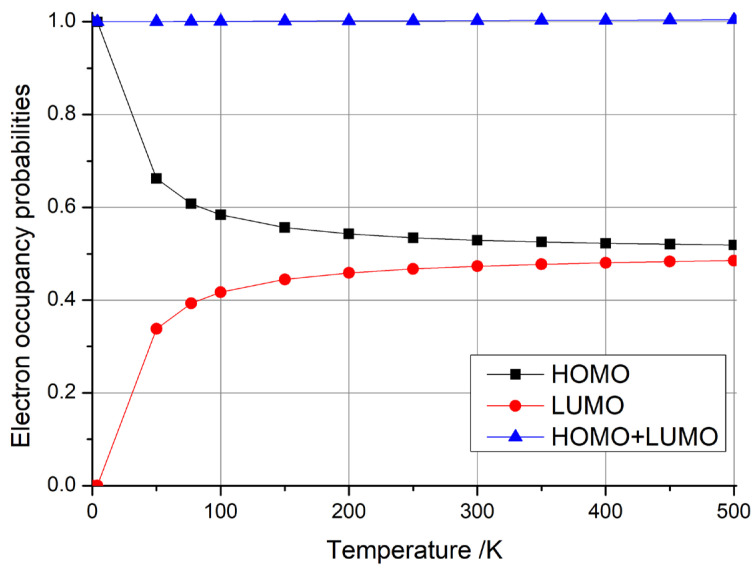
Electron occupancy probabilities of HOMO, LUMO, and HOMO + LUMO as functions of temperature after Fermi energy correction for one-dimensional carbon nanomaterial comprising (5,5) carbon nanotubes with three additional electrons.

We calculated the distribution of electron occupancy numbers at different temperatures after correcting for the Fermi energy when the (5,5) carbon nanotubes had three additional charges, as shown in Figure 5. A comparative analysis of the pre- and post-correction data, presented in Figure 3a and Figure 5, reveals that the Fermi level exerts a negligible influence on the occupancy probabilities of HOMO and LUMO up until ambient temperatures (300 K). Notably, at 500 K, a marginal 0.4% discrepancy emerges, suggesting that the Fermi energy correction can be deemed inconsequential within a normal temperature range.

### 3.2. Zero-Dimensional and Two-Dimensional Carbon Nanomaterials Containing Approximately 100 C Atoms with 0–6 Additional Electrons

**Figure 6:**
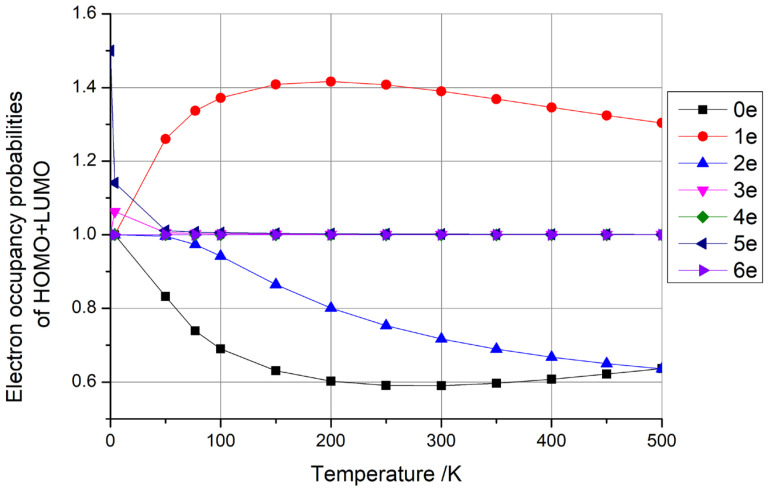
Electron occupancy probabilities of HOMO + LUMO as functions of temperature for C_100_ fullerene with 0–6 additional electrons.

**Figure 7:**
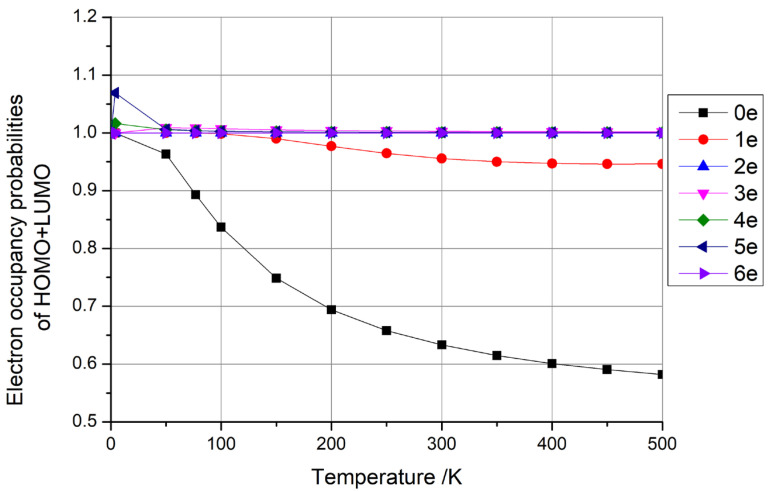
Electron occupancy probabilities of HOMO + LUMO as functions of temperature for graphene with 0–6 additional electrons.

The findings presented earlier were derived solely from one-dimensional carbon nanomaterials consisting of (5,5) carbon nanotubes with 100 C atoms. To extend our investigation, we conducted calculations for zero-dimensional carbon nanomaterials using C_100_ fullerene and two-dimensional carbon nanomaterials using square graphene, each containing approximately 100 C atoms and varying numbers of additional electrons (0–6). The outcomes of these investigations are presented in Figure 6 and Figure 7, respectively.

From Figure 6 and Figure 7, it is evident that the sum of the HOMO + LUMO occupancy probabilities remained constant at 1 when C_100_ fullerene contained four and six additional electrons, and when graphene contained two and six additional electrons. As previously discussed, such configurations hold potential for qubit fabrication. Conversely, when the sum of the HOMO + LUMO occupancy probabilities exceeded 1, the fullerene had one, three, and five additional electrons, and graphene had three, four, and five additional electrons. When the sum of the HOMO + LUMO occupancy probabilities was less than 1, the fullerene had zero and two additional electrons, and the graphene had zero and one additional electrons. The latter two scenarios do not lend themselves to qubit preparation, as per the insights garnered earlier.

### 3.3. Carbon Nanomaterials Containing 60 and 140 C Atoms with 0–6 Additional Electrons

As mentioned above, these findings were derived from carbon nanomaterials containing approximately 100 C atoms with 0–6 extra electrons. To verify the universality of the theoretical finding that low-dimensional carbon nanomaterials with specific extra electrons could be used to prepare qubits, we extended our calculations to carbon nanomaterials with varying carbon atom counts. As illustrative examples, we selected fullerenes containing 60, 100, and 140 C atoms for our computational analysis.

**Figure 8:**
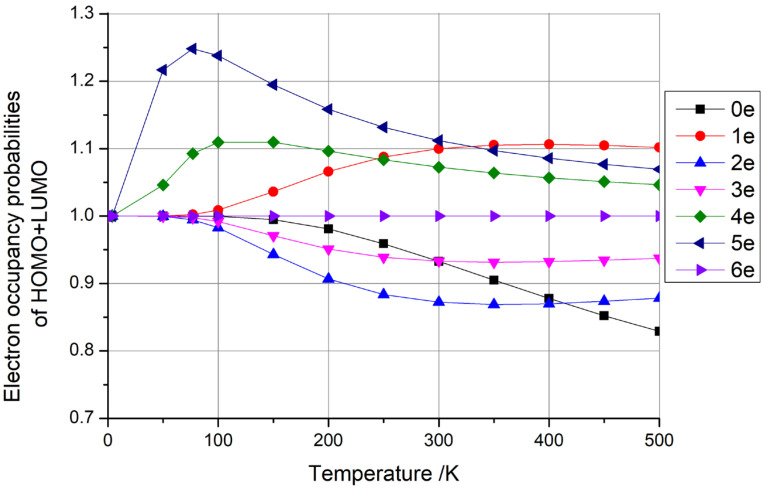
Electron occupancy probabilities of HOMO + LUMO as functions of temperature for C_60_ fullerene with 0–6 additional electrons.

**Figure 9:**
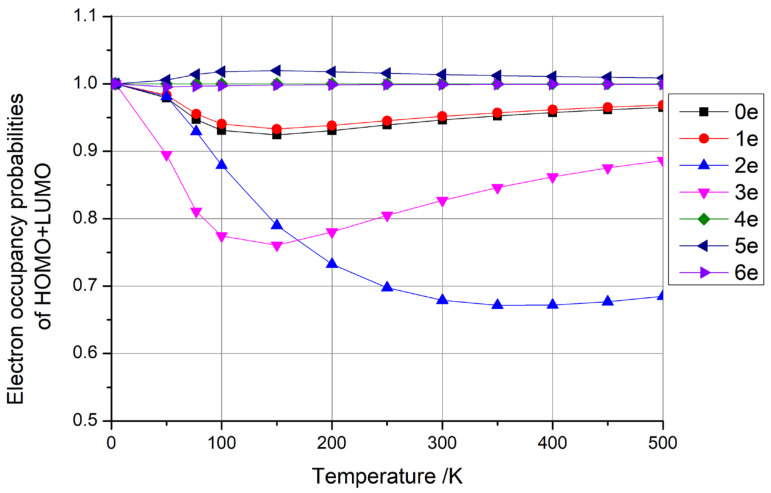
Electron occupancy probabilities of HOMO + LUMO as functions of temperature for C_140_ fullerene with 0–6 additional electrons.

Our calculations revealed intriguing trends in the HOMO + LUMO occupancy probabilities. Notably, when C_60_ fullerene possessed six extra electrons and C_140_ fullerene carried four extra electrons, the sum of these probabilities remained constant at 1, as depicted in Figure 8 and Figure 9, respectively. This observation aligns with our earlier discussion, suggesting that these specific configurations are viable candidates for qubit fabrication. Conversely, the sum of the HOMO + LUMO occupancy probabilities was greater than 1 when C_60_ had one, four, and five extra electrons and when C_140_ had five extra electrons. Additionally, the sum of the HOMO + LUMO occupancy probabilities was less than 1 when C_60_ had zero, two, and three extra electrons and when C_140_ had zero, one, two, three, and six extra electrons. Based on the rationale outlined earlier, these latter two scenarios do not offer suitable conditions for qubit preparation.

Combining all the previous findings (Table 1), it becomes evident that zero-dimensional, one-dimensional, and two-dimensional carbon nanomaterials can serve as platforms for creating qubits when they carry specific quantities of extra electrons. In other words, the feasibility of qubit preparation is independent of the type of carbon nanomaterial or the total number of carbon atoms, but critically dependent on the precise number of additional electrons, which necessitates precise calculations for identification and matching.

Furthermore, our investigations uncovered a pivotal relationship: when E_HOMO_ + E_LUMO_ = 2E_Fermi_, the sum of the occupancy probabilities of HOMO and LUMO remains constant at 1. Conversely, if the condition E_HOMO_ + E_LUMO_ < 2E_Fermi_ was met, the sum of the occupancy probabilities of HOMO + LUMO was always greater than 1. On the other hand, if the condition E_HOMO_ + E_LUMO_ > 2E_Fermi_ was met, the sum of the occupancy probabilities of HOMO + LUMO was always less than 1. Crucially, our calculations underscore that the neutral state, with no additional electrons, is unsuitable for qubit preparation. Instead, by strategically manipulating the energy-level structure of zero-dimensional, one-dimensional, and two-dimensional carbon nanomaterials through charge addition, we aimed to achieve the critical condition of E_HOMO_ + E_LUMO_ = 2E_Fermi_, which emerged as the prerequisite for qubit fabrication. This innovative strategy presents a novel pathway for qubit preparation, with the potential to significantly advance the horizons of quantum computing and its diverse applications.

### 3.4. Feasibility and Advancement Analysis of Proposed System

The proposed carbon nanomaterial-based qubit platform demonstrates both experimental feasibility and technological advancement, as supported by theoretical insights and existing experimental precedents. Below, we analyze these aspects systematically.

#### 3.4.1. Experimental Feasibility

While charge neutrality in isolated systems remains fundamental, contemporary research demonstrates viable charge modulation strategies, such as introducing dopants for controlled charge transfer, which has been experimentally validated in fullerene-based qubits [38]. The controlled charge states essential for qubit operation can be achieved via gate voltage modulation or electron beam irradiation. For instance, back-gated field-effect transistors (FETs) enable precise electron injection into carbon nanotubes or graphene, adjusting their charge density and spin polarization. Such methods have been experimentally validated in carbon nanotube transistors, where gate-controlled charge carriers exhibit single-electron resolutions [21]. Additionally, electrostatic confinement in graphene quantum dots (GQDs) allows tunable HOMO-LUMO gaps, mimicking quantum dot behavior. These techniques align with established protocols in nanoscale device fabrication, ensuring practical implementation [11].

#### 3.4.2. Material Superiority over Conventional Systems

Compared to bulk semiconductors or molecular systems, low-dimensional carbon nanomaterials offer distinct advantages:

- Quantum Confinement Effects: Discrete HOMO-LUMO energy levels in carbon nanomaterials enable precise charge and spin manipulation, unachievable in bulk semiconductors with continuous bands.
- Weak Spin–Orbit Coupling: The absence of heavy atoms in carbon systems minimizes spin decoherence, enhancing spin lifetime.
- Thermal Stability: The HOMO + LUMO occupancy remains stable up to 300 K (Figure 5), outperforming temperature-sensitive systems like diamond NV centers.
- Dimensional Flexibility: The diversity of carbon nanomaterials (0D fullerenes, 1D nanotubes, 2D graphene) allows for their tailored selection for specific quantum applications [39].

#### 3.4.3. Technological Advancement

The system introduces a novel paradigm for qubit design by leveraging temperature-dependent Fermi–Dirac statistics. Unlike optical or microwave-driven qubits (e.g., NV centers), our approach enables the thermal modulation of orbital occupancy, simplifying device architectures [29]. Furthermore, the scalability of carbon nanomaterials, facilitated by bottom-up synthesis or top-down lithography, supports large-scale qubit array integration.

#### 3.4.4. Experimental Precedents

Recent studies validate the feasibility of carbon-based quantum devices. For example, spin-polarized transport in carbon nanotubes and optically active defects in graphene demonstrate the compatibility of these materials with quantum information protocols [40]. Gate-defined quantum dots in bilayer graphene further highlight their potential for scalable qubit platforms.

#### 3.4.5. Computational Rationality

First, our calculated bandgap value of 2.76 eV for C_60_ demonstrates excellent agreement with the literature value of 2.74 eV [35]. Second, while we acknowledge that the PBE functional may underestimate the absolute values of the HOMO and LUMO energy levels, the fundamental criterion for qubit implementation (E_HOMO_ + E_LUMO_ = 2E_Fermi_) inherently relies on the relative positioning of these energy levels rather than their absolute magnitudes. In addition, it should be noted that in DFT pseudopotential methods, the absolute values of energies depend on the pseudopotential reference (e.g., vacuum level) and lack physical meaning. However, the relative energy difference (E_LUMO_ − E_HOMO_) and the alignment of E_HOMO_ + E_LUMO_ relative to 2E_Fermi_ are reference-independent. Specifically, the criterion E_HOMO_ + E_LUMO_ = 2E_Fermi_ is equivalent to E_LUMO_ − E_Fermi_ = E_Fermi_ − E_HOMO_, which depends only on the symmetry of the energy levels around the Fermi energy. This relationship remains valid regardless of the pseudopotential’s reference point. Consequently, potential underestimations by the PBE functional do not compromise the validity of our conclusions. Third, prior works on carbon-based systems have shown that GGA reliably predicts relative energy level shifts induced by doping or external charges, which are critical for qubit design [41]. Fourth, the universality of this criterion is corroborated by its applicability to all of our studied systems (0D fullerene, 1D nanotube, 2D graphene), regardless of dimensionality or atom count. This consistency across dimensionalities and systems may suggest that the relationship is intrinsic to the electronic structure of charged carbon nanomaterials and not an artifact of the computational method.

#### 3.4.6. Key Challenges, Mitigation Strategies, and Future Directions

The key challenges include environmental noise and material stability. Encapsulation with hexagonal boron nitride (hBN) or Al_2_O_3_ layers effectively suppresses charge fluctuations [42]. Directed self-assembly techniques enable the precise positioning of nanomaterials on pre-patterned substrates, addressing scalability concerns [43]. To further strengthen the work, we propose the following directions: (1) Quasiparticle Correction Analysis: Investigate quasiparticle corrections (e.g., GW approximation) for selected systems to quantify the impact of GGAs on absolute energy levels. (2) Experimental Validation: Compare GGA-derived results with experimental data (e.g., scanning tunneling spectroscopy) to verify relative energy level shifts and validate the proposed qubit design criteria.

Compared to traditional quantum systems, carbon nanomaterials exhibit unique advantages (Table 2). The carbon nanomaterial-based qubit system combines experimental accessibility, material-specific advantages, and innovative control mechanisms, positioning it as a promising candidate for next-generation quantum computing architectures.

In summary, our charged carbon nanomaterial quantum bit design demonstrates significant advantages in operational temperature tolerance and scalability. While its practical implementation currently faces technical challenges, such as interface coupling efficiency and charge state stability, we anticipate that continued advancements in material engineering and control techniques will progressively address these limitations. Consequently, these quantum systems are poised to fulfill specialized roles in quantum sensing and information processing applications where their unique charge-tunable characteristics offer distinct advantages over conventional qubit architectures.

## 4. Conclusions

We demonstrate through first-principles calculations that low-dimensional carbon nanomaterials—including fullerenes, nanotubes, and graphene—exhibit tunable HOMO-LUMO occupancy probabilities under controlled charge states. When E_HOMO_ + E_LUMO_ = 2E_Fermi_, the combined occupancy remains temperature-invariant at unity, fulfilling the fundamental requirement for qubit operation. This behavior is universal across materials with varying dimensionalities and atom counts, provided precise electron doping is achieved. Crucially, the system’s experimental feasibility is underscored by established techniques such as electrostatic gating and quantum dot confinement, while its advantages over conventional qubit platforms (e.g., room-temperature stability, scalability, and reduced decoherence) position it as a transformative candidate for quantum technologies. Challenges such as environmental noise and interfacial stability can be mitigated via encapsulation and directed assembly strategies. This work not only expands the toolkit for quantum information science but also paves the way for scalable, carbon-based quantum architectures.

## Figures and Tables

**Table 1:** Electron occupancy probabilities of HOMO + LUMO with 0–6 additional electrons in different models.

Model/Charge	0e	1e	2e	3e	4e	5e	6e
C_60_	<1	>1	<1	<1	>1	>1	=1
C_100_	<1	>1	<1	>1	=1	>1	=1
C_140_	<1	<1	<1	<1	=1	>1	<1
CNT	<1	>1	<1	=1	=1	=1	<1
Graphene	<1	<1	=1	>1	>1	>1	=1

**Table 2:** Key parameter comparison between carbon nanomaterials and other quantum systems.

Parameter/System	Carbon Nanomaterials	NV Centers	Semiconductor Quantum Dots
Regulation Method	Charge Injection/Temperature	Optical/Microwave	Electric Field/Magnetic Field
Scalability	High	Low	Medium
Coherence Time	Long	Longest	Shorter

## Data Availability

The original contributions presented in this study are included in the article. Further inquiries can be directed to the corresponding author.
